# Quantum Positioning Scheme Based on Microwave–Optical Entanglement

**DOI:** 10.3390/s24237712

**Published:** 2024-12-02

**Authors:** Qiang Miao, Dewei Wu

**Affiliations:** Laboratory of Advanced Navigation Technology, Information and Navigation College, Air Force Engineering University, Xi’an 710049, China; wudewei74609@126.com

**Keywords:** microwave–optical entanglement, nano-cavity electro-opto-mechanic converter, quantum positioning system, quantum navigation

## Abstract

Microwaves exhibit superior performance in free-space transmission compared to optical waves, primarily due to their ability to penetrate fog and experience lower losses in the Earth’s atmosphere. Based on microwave–optical entanglement prepared by nano-cavity electro-opto-mechanic converters, we propose a scheme of a quantum positioning system using the distance-based positioning method. Principles of microwave–optical entanglement preparation and our QPS scheme are introduced in detail. The entanglement feature, system stability and positioning feature of the scheme are analyzed after simulations. Furthermore, we delve into the impact of key parameters, such as transmissivity and photon conversion efficiency, on positioning. Notably, the entanglement degrees for both microwave–optic entanglement at the transmitter and optic–optic entanglement at the receiver surpass one, affirming the efficiency of the scheme in preparing and maintaining entanglement. When transmissivity in beam-splitter models of both ground stations equals 0.5, our scheme achieves a minimal positioning error of $6.4\times 10^{-7}m^{2}$ under ideal conditions. Additionally, we map out traces of a plane through continuous positioning using our scheme. These results demonstrate the theoretical efficiency and robustness of our proposed approach.

## 1. Introduction

Quantum entanglement is the core of quantum mechanics [1]; it is applied in various fields and results in remarkable performance promotions [2]. These promotions originate from non-classical features of entanglement.

Positioning is one of the most significant functions of a navigation system, which requires considerable precision. Extensive endeavors have been undertaken to integrate quantum sources into navigation positioning systems, aiming to enhance clock synchronization [3], positional accuracy, anti-interference capabilities, and various other metrics. These innovative applications collectively constitute the quantum positioning system (QPS) [4,5,6]. Primarily, these investigations leverage the optical frequency band due to its ease in quantum entanglement preparation and detection, which is notably challenging in the microwave domain. However, because microwave frequencies can penetrate fog and are less lossy in the Earth’s atmosphere compared to optic waves, microwaves are superior to optical waves in free-space transmission [7], making them preferable for many navigation devices designed to operate in the microwave spectrum.

Recent advancements in nano-cavity opto-mechanical systems introduce an intriguing method to combine the advantages of microwaves and optical waves by entangling them both with a mechanical resonator mode. This technology is referred to as the nano-cavity electro-opto-mechanical (EOM) converter [8,9]. This allows the transformation from tough microwave photon detection to convenient optical photon detection while retaining the microwave transmission feature.

In this paper, we introduce a QPS scheme that leverages microwave–optical (M–O) entanglement generated by EOM converters. We elucidate the fundamental principles behind M–O entanglement preparation and our proposed QPS scheme and conduct simulations under appropriate parameter settings. A comprehensive analysis is provided to examine the quantum entanglement characteristics and precision of our scheme. Furthermore, we delve into the various factors that can impact the performance of our system.

## 2. EOM Converter and M–O Entanglement

### 2.1. Cavity Optomechanical Systems and EOM Converter

Cavity optomechanical systems [9,10,11,12,13] have attracted considerable attention in the past few years due to their potential applications in quantum information technology. Earlier research has achieved dramatic progress in electromechanically or optomechanically induced transparency (EMIT/OMIT) [14,15,16], quantum mechanics in macroscopic objects [17,18,19,20], cooling [21,22,23,24,25,26], optical filters [27], quantum sensors [10,28,29], etc. Furthermore, nano-mechanical resonators can be coupled to the microwave and optical cavities simultaneously to form a nano-cavity opto-mechanical system called an EOM converter. This can therefore entangle [30,31] or coherently convert [32,33] the photons in the microwave and optical regions.

**Figure 1:**
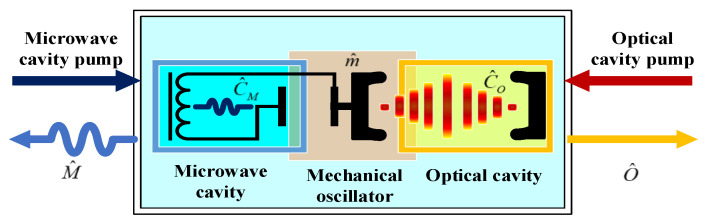
The schematic of EOM converter.

The EOM converter primarily comprises three components: a microwave cavity equipped with LC circuits, an optical Fabry–Perot cavity, and a mechanical oscillator. The mechanical oscillator serves as both the movable plate of the capacitor in the LC circuit and the movable mirror of the optical cavity [34,35,36,37,38]. Refer to Figure 1 for the schematic representation of the EOM converter.

Hence, the microwave photons and the optical photons are both coupled with mechanical oscillator phonons. In other words, the microwave cavity mode is coupled with the optical cavity mode. For simplicity, we list the parameters and their denotations in the EOM converter in Table 1 [38,39,40,41,42,43].

The detuning of each cavity can be denoted as $\Delta_{j}=\omega_{j}-\omega_{d,j}$, where the subscript j=M,O represents the specific parameter for either the microwave or optical wave throughout the paper. For brevity, we will omit further repetition of the subscript j=M,O in subsequent references.

Each cavity mode can be characterized as the sum of steady-state amplitude and the quantum fluctuation around it: ${\hat{C}}_{j}=C_{j}+\delta {\hat{C}}_{j}$. The coupling rate between the cavities and the mechanical resonator is denoted as $G_{j}=g_{j}\sqrt{C_{j}}$, where $g_{j}$ represents the single photon–phonon coupling rate and $C_{j}$ corresponds to the steady-state amplitude of the respective cavity mode. Provided that the frequency detuning of the cavity fulfills the condition $\Delta_{M}=-\Delta_{O}=\omega_{m}$, and after applying linearization and rotation approximations, the free Hamiltonian of the EOM converter in the interaction picture can be formulated as shown in Equation (1) [38].

(1) $$H=\hbar G_{M}(\delta {\hat{C}}_{M}{\hat{m}}^{†}+\hat{m}\delta {{\hat{C}}_{M}}^{†})+\hbar G_{O}(\delta {\hat{C}}_{O}\hat{m}+{\hat{m}}^{†}\delta {{\hat{C}}_{O}}^{†})$$

Equation (1) shows the nature of the EOM converter coupling the M–O entanglement. The first term is a beam-splitter-like term, which exhibits the entanglement between microwave cavity mode and mechanical oscillator mode, while the second term is a parametric down-conversion-like term that reveals the entanglement between optical cavity mode and mechanical oscillator mode. If the microwave–mechanical coupling rate $G_{M}^{2}/\kappa_{M}>R_{d}$ and the optical–mechanical coupling rate $G_{O}^{2}/\kappa_{O}>R_{d}$, where $R_{d}$ is the decoherence rate of the mechanical resonator, entanglement would be transmitted to the output of each cavity. Thus, the propagating modes are entangled.

### 2.2. Propagating Modes and Entanglement Degree

Utilizing quantum Langevin equations, the propagating modes can be expressed as [38]

(2) $$\hat{M}=A_{M}\delta {\hat{C}}_{M,intra}-B\delta {\hat{C}}_{O,intra}^{†}-C_{M}\delta {\hat{C}}_{m}$$

(3) $$\hat{O}=B\delta {\hat{C}}_{M,intra}^{†}+A_{O}\delta {\hat{C}}_{O,intra}-C_{O}\delta {\hat{C}}_{m}^{†}$$

where $\hat{M}$ and $\hat{O}$ are the propagating microwave and optical field, respectively; $\delta {\hat{C}}_{j,intra}$ denotes the intracavity quantum fluctuation; and $\delta {\hat{C}}_{m}$ signifies quantum Brownian noise arising from the thermally excited phonons within the mechanical oscillator. Furthermore, $A_{j}$, B, $C_{j}$ are parameters related with cooperativity parameters $\Gamma_{j}=G_{j}^{2}/\kappa_{j}\gamma$, which represents the cooperative interactions between different modes. Detailed expressions of $A_{j}$, B, $C_{j}$ can be found in Appendix A.

Another important issue is the quantitative assessment of entanglement. To address this, we utilize the entanglement degree, which is based on the average photon number of the propagating modes and their correlation. Considering the temperature of the electro-optic modulator (EOM) as $T_{E}$, we can determine the average photon or phonon number of the quantum fluctuations in Equations (2) and (3) at their respective frequencies using Planck’s laws, which read [38]

(4) $$\left\{\begin{array}{l} {\bar{n}}_{M}=\frac{1}{e^{\hbar \omega_{M}/k_{B}T_{E}}-1}\\ {\bar{n}}_{O}=\frac{1}{e^{\hbar \omega_{O}/k_{B}T_{E}}-1}\\ {\bar{n}}_{m}=\frac{1}{e^{\hbar \omega_{m}/k_{B}T_{E}}-1} \end{array}\right.$$

The average photon number of the propagating modes and their correlation can be expressed as [38]

(5) $${\bar{N}}_{M}=\langle {\hat{M}}^{†}\hat{M}\rangle ={\left|A_{M}\right|}^{2}{\bar{n}}_{M,T_{E}}+{\left|B\right|}^{2}\left({\bar{n}}_{O,T_{E}}+1\right)+{\left|C_{M}\right|}^{2}{\bar{n}}_{m,T_{E}}$$

(6) $${\bar{N}}_{O}=\langle {\hat{O}}^{†}\hat{O}\rangle ={\left|B\right|}^{2}\left({\bar{n}}_{M,T_{E}}+1\right)+{\left|A_{O}\right|}^{2}{\bar{n}}_{O,T_{E}}+{\left|C_{O}\right|}^{2}\left({\bar{n}}_{m,T_{E}}+1\right)$$

(7) $$\langle \hat{M}\hat{O}\rangle =A_{M}B({\bar{n}}_{M,T_{E}}+1)-BA_{O}{\bar{n}}_{O,T_{E}}+C_{M}C_{O}({\bar{n}}_{m,T_{E}}+1)$$

whose detail deductions can be found in Appendix B.

#### 2.2.1. Metric of Induced Correlation

If $\hat{M}$ and $\hat{O}$ are merely two classical random complex variables, then a joint Gaussian state is classified as a classical state only if it satisfies the following relationship, as outlined in [38]:

(8) $$\langle \left|MO^{*}\right|\rangle \leq \langle \left|M\right|\rangle \langle \left|O^{*}\right|\rangle =\sqrt{{\bar{N}}_{M}}\sqrt{{\bar{N}}_{O}}$$

When Equation (8) is violated, the joint Gaussian state lacks a classical counterpart, suggesting that the hybrid state composed of a microwave and an optical output is indeed a quantum state. The cavity electro-opto-mechanic converter produces hybrid entanglement, and the violation of Equation (8) serves as the criterion for identifying such hybrid entanglement [38].

(9) $$\langle \left|\hat{M}\hat{O}\right|\rangle =\left|\langle \hat{M}\hat{O}\rangle \right|>\sqrt{{\bar{N}}_{M}}\sqrt{{\bar{N}}_{O}}$$

We can discuss the entanglement degree quantitatively by induced correlation as [38]:

(10) $$\varepsilon =\frac{\left|\langle \hat{M}\hat{O}\rangle \right|}{\sqrt{{\bar{N}}_{M}{\bar{N}}_{O}}}$$

As previously mentioned, entanglement among propagating modes can only occur if certain conditions involving $G_{M}^{2}/\kappa_{M}>R_{d}$ and $G_{O}^{2}/\kappa_{O}>R_{d}$ are met. Here, $R_{d}=\gamma {\bar{n}}_{m}$ represents the decoherence rate of the mechanical resonator. In terms of the induced correlation ε, the entanglement criterion can be stated as follows: if the value of ε exceeds 1, entanglement exists between the propagating modes, with the strength of quantum correlation and the degree of entanglement intensifying as the value increases. Conversely, if this condition is not satisfied, decoherence and other factors will prevent the formation of entanglement.

In fact, we subsequently employ the induced correlation ε to quantify entanglement in optical coincidence measurements in this paper.

#### 2.2.2. Metric of Logarithmic Negativity

The quantification of microwave–optic entanglement can also be represented by logarithmic negativity, which originates from the positive partial transposition (PPT) criterion and is currently a widely used entanglement metric method. The covariance matrix of microwave–optic entanglement V(ω) can be expressed as [41]

(11) $$V(\omega )=\left(\begin{array}{cccc} V_{11} & 0 & V_{13} & 0\\ 0 & V_{11} & 0 & -V_{13}\\ V_{13} & 0 & V_{33} & 0\\ 0 & -V_{13} & 0 & V_{33} \end{array}\right)$$

This is the covariance matrix of vector $\nu ={\left[{\hat{X}}_{M}(\omega ),{\hat{Y}}_{M}(\omega ),{\hat{X}}_{O}(\omega ),{\hat{Y}}_{O}(\omega )\right]}^{T}$, which is composed of the orthogonal components of microwave and optical modes in the frequency domain. Each element can be obtained from

(12) $$\delta (\omega +\omega^{\prime })V_{ij}(\omega )=\frac{1}{2}\langle \nu_{i}(\omega )\nu_{j}(\omega^{\prime })+\nu_{j}(\omega^{\prime })\nu_{i}(\omega )\rangle$$

Logarithmic negativity can be represented by the minimum transposed symplectic eigenvalue $\xi^{-}$ of the covariance matrix V(ω) as follows [41]:

(13) $$E_{N}=\max\{0,-\log(2\xi^{-})\}$$

$\xi^{-}$ can be solved by the following equation [41]:

(14) $$\xi^{-}={\left(V_{11}^{2}+V_{33}^{2}+2V_{13}^{2}-\sqrt{{\left(V_{11}^{2}-V_{33}^{2}\right)}^{2}+4V_{13}^{2}{\left(V_{11}+V_{33}\right)}^{2}}\right)}^{1/2}/\sqrt{2}$$

$E_{N}>0$ means that the output exhibits microwave–optic entanglement, and a larger value of $E_{N}$ corresponds to a stronger degree of entanglement. Furthermore, it can be inferred from the calculation that when $E_{N}=0$ and ε=1, they are consistent on the boundary of entanglement determination.

#### 2.2.3. Comparison of Two Metrics: $E_{N}$ and ε

**Figure 2:**
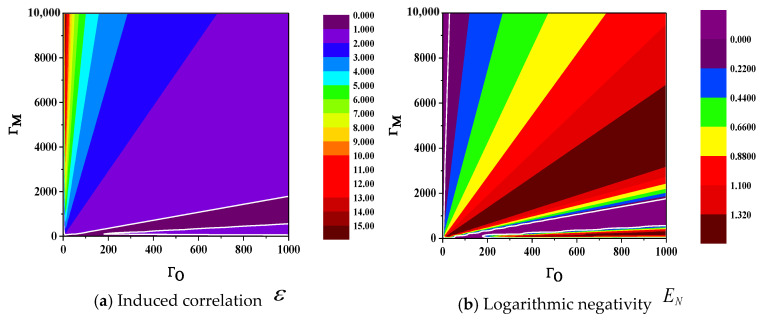
The relationship between the entanglement degree ε and $E_{N}$ of the output modes and the cooperativity parameters $\Gamma_{M}$, $\Gamma_{O}$ of cavity electro-optic converters.

The two metrics are employed to ascertain the presence of entanglement via ε>1 or $E_{N}>0$. We can analyze the relationship between induced correlation ε, logarithmic negativity $E_{N}$, and cooperativity parameters $\Gamma_{M}$ and $\Gamma_{O}$ through numerical simulation. As shown in Figure 2a,b, the white line in the figure represents their thresholds (ε=1 or $E_{N}=0$).

As depicted in Figure 2, within the parameter space defined by the cooperativity parameters $\Gamma_{M}$ and $\Gamma_{O}$, the varying trends of ε and $E_{N}$ exhibit distinct behaviors as the those parameters change. To a certain extent, we can conclude that the values of ε (or $E_{N}$) are almost equal within a small sector centered at point (0, 0) of the coordinate system. For both ε and $E_{N}$, their values within the sector have a certain relationship with the angle between the sector and $\Gamma_{O}$-axis. In Figure 2a, the value of ε approximately monotonically increases with the angle. In Figure 2b, as the angle increases, the value of $E_{N}$ exhibits two maxima, particularly within an extremely narrow sector where the angle is very small and close to the $\Gamma_{O}$-axis, where there is a moment of maximum value. This is the primary distinction between the two metrics ε and $E_{N}$, and the specific reasons lie in their respective functional relationships with $\Gamma_{M}$ and $\Gamma_{O}$.

As shown in Figure 2a,b, the thresholds for determining whether it is an entanglement of these two metrics coincide (when ε=1, $E_{N}=0$), and the regions distinguished as entangled (when $E_{N}>0$, ε>1) and non-entangled by these thresholds exhibit substantial congruence. Therefore, it can be affirmed that at least in the space defined by $\Gamma_{M}$ and $\Gamma_{O}$, the two metrics ε and $E_{N}$ are equivalent as entanglement criteria. However, there are also differences in their specific value within the entangled region, stemming from variations in the conditional constraints and sensitivity to specific parameter changes, which influence their ability to discern entanglement. Additionally, the absolute numerical changes of ε are more pronounced compared to those of $E_{N}$, making it more convenient for observation and indicating that this metric has superior discrimination capabilities.

## 3. QPS Scheme Utilizing EOM Converters

### 3.1. Principle of Distance-Based Positioning

**Figure 3:**
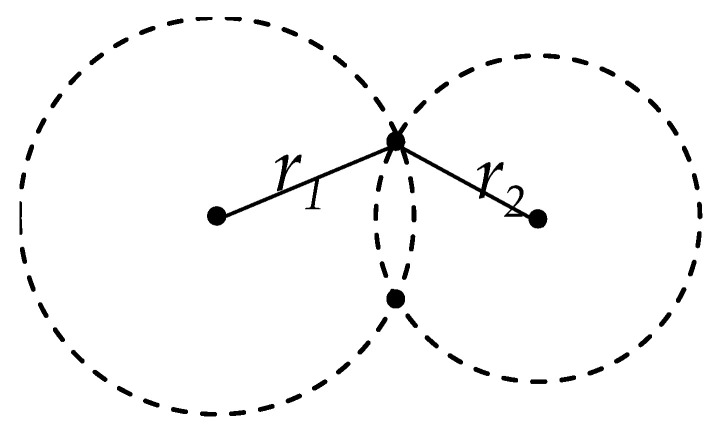
Principle of distance-based positioning.

Positioning can be realized through various methods [35,36], and one of them is circle–circle positioning. This method relies on the distance measurements between the target and navigation ground stations, as shown in Figure 3. Once these distances are determined, circles are drawn with each navigation ground station as the center and the corresponding distance as the radius. The position of the target is then located at one of the intersection points of these circles. Consequently, the accuracy of circle–circle positioning hinges on precise distance measurements between the target and the ground stations.

### 3.2. QPS Scheme Based on EOM Converters

**Figure 4:**
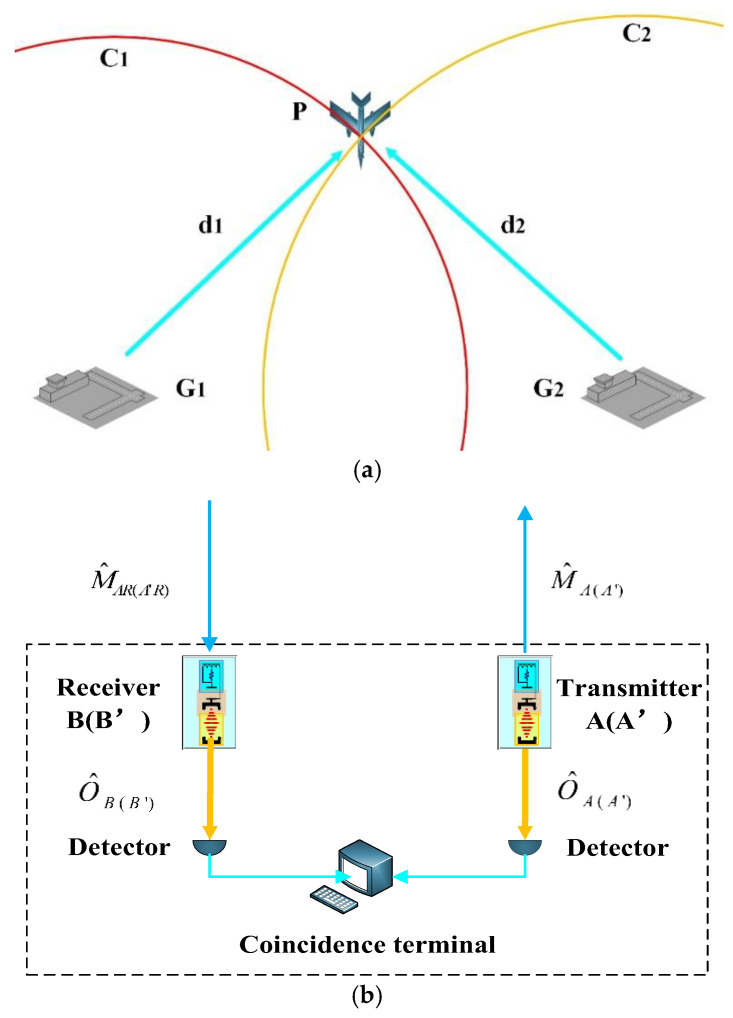
QPS scheme based on EOM converters. (**a**) Circle–circle positioning; (**b**) Ground station setup.

Utilizing EOM converters and M–O entanglement, we introduce a QPS scheme that employs circle–circle positioning. This QPS scheme involves two distance measurement processes, similar to the microwave quantum illumination proposed by Shabir Barzanjeh [38]. The schematic illustration of our proposed scheme is presented in Figure 4.

#### 3.2.1. Transmission

As illustrated in Figure 4, in our QPS scheme, we utilize G1 and G2, assuming that the plane P and both ground stations are accurately synchronized in terms of time reference. Each ground station comprises a pair of identical EOM converters, with one serving as the transmitter and the other as the receiver. Ground station G1 includes transmitter A and receiver B, while G2 includes transmitter A′ and receiver B′. Both A and A′ independently and simultaneously prepare M–O (microwave–optical) entanglement. Subsequently, the optical outputs ${\hat{O}}_{A}$ and ${\hat{O}}_{A^{\prime }}$ are retained within their respective ground stations, while the microwave outputs ${\hat{M}}_{A}$ and ${\hat{M}}_{A^{\prime }}$ are transmitted through lossy free space towards the plane P. The expressions for the propagating modes can be readily derived using Equations (15)–(18) [38].

(15) $${\hat{M}}_{A}=A_{M}\delta {\hat{C}}_{MA,intra}-B\delta {\hat{C}}_{OA,intra}^{†}-C_{M}\delta {\hat{C}}_{mA}$$

(16) $${\hat{O}}_{A}=B\delta {\hat{C}}_{MA,intra}^{†}+A_{O}\delta {\hat{C}}_{OA,intra}-C_{O}\delta {\hat{C}}_{mA}^{†}$$

(17) $${\hat{M}}_{A^{\prime }}=A_{M}\delta {\hat{C}}_{MA^{\prime },intra}-B\delta {\hat{C}}_{OA^{\prime },intra}^{†}-C_{M}\delta {\hat{C}}_{mA^{\prime }}$$

(18) $${\hat{O}}_{A^{\prime }}=B\delta {\hat{C}}_{MA^{\prime },intra}^{†}+A_{O}\delta {\hat{C}}_{OA^{\prime },intra}-C_{O}\delta {\hat{C}}_{mA^{\prime }}^{†}$$

#### 3.2.2. Reception

Regarding the impacts that microwave signals encounter during transmission through free space, including background quantum fluctuations, signal loss, and absorption by the plane’s surface, we can employ a beam-splitter model to address them collectively. The transmissivity of the beam-splitter can be used to represent these effects. Notably, separate beam-splitter models must be constructed for the microwave signals ${\hat{M}}_{A}$ and ${\hat{M}}_{A^{\prime }}$, as they may undergo different transmission distances and environmental conditions. We denote the transmissivities of these beam-splitter models as $\eta_{1}$ and $\eta_{2}$, respectively. When microwave signals encounter the plane, a portion of them is absorbed by the surface, while the rest is reflected back to the ground stations, which is denoted as ${\hat{M}}_{AR}$ and ${\hat{M}}_{A^{\prime }R}$. Considering that the background temperature is $T_{B}$, the background quantum fluctuations can be denoted as $\delta {\hat{C}}_{B,T_{B}}$, with an average photon number of ${\bar{n}}_{B,T_{B}}=\frac{1}{e^{\hbar \omega_{M}/k_{B}T_{B}}-1}$.

Hence, the reflected signals ${\hat{M}}_{AR}$ and ${\hat{M}}_{A^{\prime }R}$ can be expressed as follows [38]:

(19) $${\hat{M}}_{AR}=\sqrt{\eta_{1}}{\hat{M}}_{A}+\sqrt{1-\eta_{1}}\delta {\hat{C}}_{B,T_{B}}$$

(20) $${\hat{M}}_{A^{\prime }R}=\sqrt{\eta_{2}}{\hat{M}}_{A^{\prime }}+\sqrt{1-\eta_{2}}\delta {\hat{C}}_{B,T_{B}}$$

After being received, ${\hat{M}}_{AR}$ and ${\hat{M}}_{A^{\prime }R}$ are upconverted into optical outputs ${\hat{O}}_{B}$ and ${\hat{O}}_{B^{\prime }}$ by B and B′, respectively [38].

(21) $${\hat{O}}_{B}=B{\hat{M}}_{AR}^{†}+A_{O}\delta {\hat{C}}_{OB,intra}-C_{O}\delta {\hat{C}}_{mB}^{†}$$

(22) $${\hat{O}}_{B^{\prime }}=B{\hat{M}}_{A^{\prime }R}^{†}+A_{O}\delta {\hat{C}}_{OB^{\prime },intra}-C_{O}\delta {\hat{C}}_{mB^{\prime }}^{†}$$

where $\delta {\hat{C}}_{OB,intra}$/$\delta {\hat{C}}_{OB^{\prime },intra}$ and $\delta {\hat{C}}_{mB}$/$\delta {\hat{C}}_{mB^{\prime }}$ are counterparts of $\delta {\hat{C}}_{OA,intra}$/$\delta {\hat{C}}_{OA^{\prime },intra}$ and $\delta {\hat{C}}_{mA}$/$\delta {\hat{C}}_{mA^{\prime }}$ in the respective receivers.

#### 3.2.3. Coincidence Measurement

Once ${\hat{O}}_{B}$ and ${\hat{O}}_{B^{\prime }}$ are output, we conduct a Hanbury Brown–Twiss (HBT) pattern coincidence measurement for ${\hat{O}}_{B}$-${\hat{O}}_{A}$ or ${\hat{O}}_{B^{\prime }}$-${\hat{O}}_{A^{\prime }}$ at the respective ground station to obtain the second-order coherence function as [39]

(23) $$g^{\left(2\right)}\left(\tau_{1}\right)=\frac{\langle {{\hat{O}}_{A}}^{†}{\hat{O}}_{A}{\hat{O}}_{B}^{†}{\hat{O}}_{B}\rangle }{\langle {{\hat{O}}_{A}}^{†}{\hat{O}}_{A}\rangle \langle {\hat{O}}_{B}^{†}{\hat{O}}_{B}\rangle }=\frac{\langle {\hat{n}}_{OA}{\hat{n}}_{OB}\rangle }{{\bar{N}}_{OA}{\bar{N}}_{OB}}$$

(24) $$g^{\left(2\right)}\left(\tau_{2}\right)=\frac{\langle {{\hat{O}}_{A^{\prime }}}^{†}{\hat{O}}_{A^{\prime }}{\hat{O}}_{B^{\prime }}^{†}{\hat{O}}_{B^{\prime }}\rangle }{\langle {{\hat{O}}_{A^{\prime }}}^{†}{\hat{O}}_{A^{\prime }}\rangle \langle {\hat{O}}_{B^{\prime }}^{†}{\hat{O}}_{B^{\prime }}\rangle }=\frac{\langle {\hat{n}}_{OA^{\prime }}{\hat{n}}_{OB^{\prime }}\rangle }{{\bar{N}}_{OA^{\prime }}{\bar{N}}_{OB^{\prime }}}$$

where ${\hat{n}}_{OA}$, ${\hat{n}}_{OB}$, ${\hat{n}}_{OA^{\prime }}$ and ${\hat{n}}_{OB^{\prime }}$ denote the photon number operators of ${\hat{O}}_{A}$, ${\hat{O}}_{B}$, ${\hat{O}}_{A^{\prime }}$ and ${\hat{O}}_{B^{\prime }}$ respectively; $\tau_{1}$ and $\tau_{2}$ are free-space transmission time intervals of microwave signals transmitted from G1 and G2. Meanwhile, the maximum second-order correlation function of the entanglement state reads [40]

(25) $$g_{\max}^{\left(2\right)}\left(\tau \right)=1+\exp(-\delta^{2}\tau^{2})$$

where δ is the spectral linewidth. Once the maximum of the second-order coherence function is obtained, we can determine the transmission time intervals $\tau_{1}$ and $\tau_{2}$. Consequently, we can estimate the approximate distances between the plane and G1/G2, respectively, by

(26) $$d_{1}=\frac{1}{2}c\tau_{1}$$

(27) $$d_{2}=\frac{1}{2}c\tau_{2}$$

where *c* denotes the propagating speed of microwaves in free space. Once $d_{1}$ and $d_{2}$ are determined, circles centered on G1 and G2 with radii $d_{1}$ and $d_{2}$ (denoted as C1 and C2) are consequently established. These circles intersect at two points, but only one of them represents the actual position of the plane. By utilizing additional known information, we can readily discard the false intersection.

## 4. Results and Discussions

To evaluate the performance of our QPS scheme, we assume experimentally achievable parameters for our simulations. The parameter settings for the EOM converters are outlined in Table 2 below.

Furthermore, the quality factor *Q* and mass *m* of the mechanical resonator are set as $30\times 10^{3}$ and 10 ng. As for the environment settings, we assume that $T_{E}=30$ mK, $T_{B}=293$ K. The distance between G1 and G2 is 600 m.

### 4.1. Entanglement Feature

We can readily calculate the entanglement degrees of the pairs ${\hat{M}}_{A}$-${\hat{O}}_{A}$, ${\hat{M}}_{A^{\prime }}$-${\hat{O}}_{A^{\prime }}$, ${\hat{O}}_{B}$-${\hat{O}}_{A}$ and ${\hat{O}}_{B^{\prime }}$-${\hat{O}}_{A^{\prime }}$ using the same method as in Equations (5)–(8), and we denote them as ε (for ${\hat{M}}_{A}$-${\hat{O}}_{A}$, and ${\hat{M}}_{A^{\prime }}$-${\hat{O}}_{A^{\prime }}$), and $\varepsilon_{R}$ (for ${\hat{O}}_{B}$-${\hat{O}}_{A}$ and ${\hat{O}}_{B^{\prime }}$-${\hat{O}}_{A^{\prime }}$) respectively. Given the parameter settings in this paper, we obtain ε=1.4950>1, indicating that the entanglements between ${\hat{M}}_{A}$-${\hat{O}}_{A}$, and ${\hat{M}}_{A^{\prime }}$-${\hat{O}}_{A^{\prime }}$ are well-prepared. This is further confirmed by the values $G_{M}^{2}/\kappa_{M}=9.493\times 10^{5}$, $G_{O}^{2}/\kappa_{O}=1.254\times 10^{5}$ and $R_{d}=5.298\times 10^{4}$, which satisfy the conditions $G_{M}^{2}/\kappa_{M}>R_{d}$ and $G_{O}^{2}/\kappa_{O}>R_{d}$ well. Additionally, we obtain $\varepsilon_{R}=1.0301>1$, indicating that ${\hat{O}}_{B}$-${\hat{O}}_{A}$ and ${\hat{O}}_{B^{\prime }}$-${\hat{O}}_{A^{\prime }}$ are also entangled individually. This demonstrates that the entanglement feature is preserved after transmission through free space.

**Figure 5:**
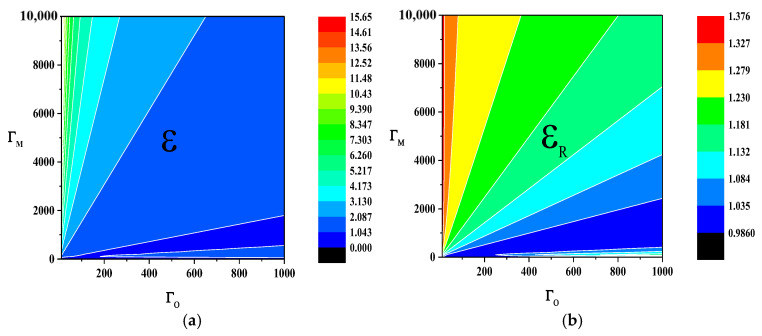
(**a**) ε versus $\Gamma_{M}$ and $\Gamma_{O}$; (**b**) $\varepsilon_{R}$ versus $\Gamma_{M}$ and $\Gamma_{O}$.

Although quantum entanglements in G1 and G2 are independent, ε of them appear to be same in terms of the cooperativity parameters $\Gamma_{M}$ and $\Gamma_{O}$, because the main parameter settings of EOM converters are the same. Given that $\eta_{1}=\eta_{2}=0.5$, ε exhibits the same pattern versus $\Gamma_{M}$ and $\Gamma_{O}$ with $\varepsilon_{R}$. They are depicted in Figure 5. 

It is evident from Figure 5 that ε>1 holds true in the majority of cases involving $\Gamma_{M}$ and $\Gamma_{O}$, which indicates that the microwave–optical entanglement, generated by the EOM under the conditions specified in this paper, exhibits a high degree of stability.

### 4.2. System Stability

A stable cavity electro-opto-mechanic converter is the basis for quantum positioning. Its stability can be determined by the Routh Hurwitz criterion, which is specifically expressed as [33,38]

(28) $$\kappa_{M}\Gamma_{M}-\kappa_{O}\Gamma_{O}>K\max\left(\kappa_{M}-\kappa_{O},\frac{{\kappa_{O}}^{2}-{\kappa_{M}}^{2}}{2\gamma_{m}+\kappa_{M}+\kappa_{O}}\right)$$

where $K=\frac{\Gamma_{M}}{1+\kappa_{O}/\kappa_{M}}+\frac{\Gamma_{O}}{1+\kappa_{M}/\kappa_{O}}$. For the convenience of quantifying system stability, the ratio stability *S* is defined as

(29) $$S=\frac{\kappa_{M}\Gamma_{M}-\kappa_{O}\Gamma_{O}}{K\max\left(\kappa_{M}-\kappa_{O},\frac{{\kappa_{O}}^{2}-{\kappa_{M}}^{2}}{2\gamma_{m}+\kappa_{M}+\kappa_{O}}\right)}$$

In Equation (29), S=1 represents the stability boundary of the system, which is considered stable when S>1; otherwise, it is in an unstable state.

**Figure 6:**
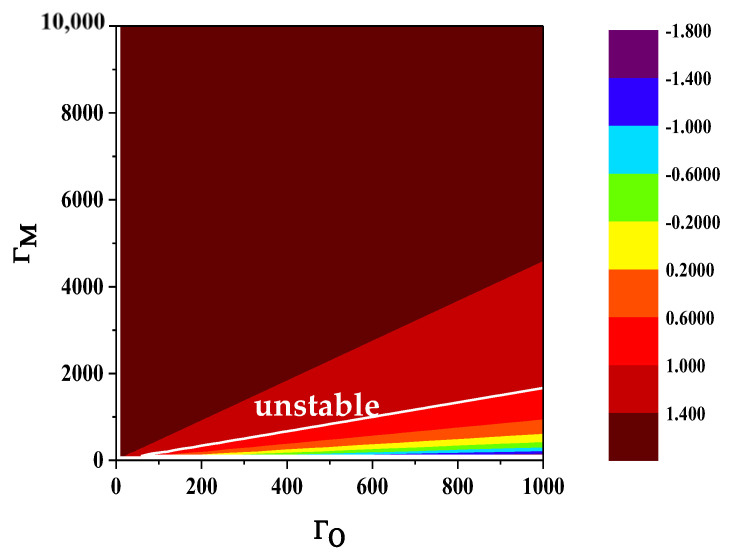
The relationship between S and the cooperativity parameters.

Based on Equation (29), the relationship between S and the cooperativity parameters $\Gamma_{M}$ and $\Gamma_{O}$ can be visualized as depicted in Figure 6, with the white line denoting the threshold value. A comparison of Figure 2 and Figure 6 reveals that the threshold for the entanglement criterion coincides with the system’s stability threshold. Furthermore, the area where entanglement does not occur corresponds to the unstable operational zone. This suggests that within the domain defined by the cooperativity parameters $\Gamma_{M}$ and $\Gamma_{O}$, ε and $E_{N}$ can serve as effective measures of the entanglement degree while simultaneously characterizing the stability of the system. From this perspective, both ε and $E_{N}$ are highly suitable metrics for conducting quantitative analyses of hybrid entanglement in cavity electro-optic-mechanic converters.

In summary, in the space defined by the cooperativity parameters, the cavity electro-opto-mechanic converter can effectively prepare hybrid microwave–optic entanglement in the vast majority of cases, and the system remains in a stable state when hybrid entanglement exists. This theoretically verifies the effectiveness and robustness of the cavity electro-opto-mechanic converter.

### 4.3. Quantum Positioning Feature

#### 4.3.1. Performance of Positioning

**Figure 7:**
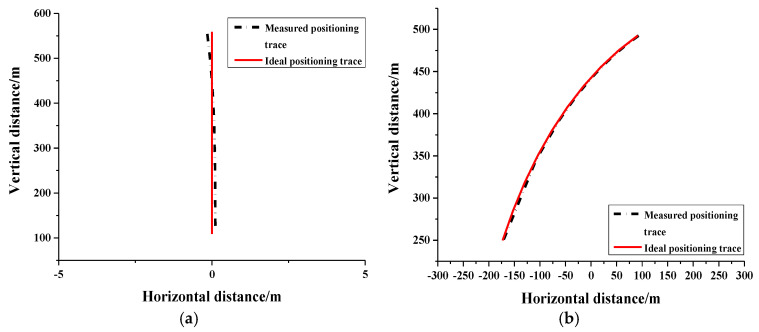
(**a**) Traces of flying along with middle line between ground stations; (**b**) Traces of flying around one ground station.

For clarity and ease of understanding, we have established a rectangular coordinate system with its transverse axis connecting G1 and G2. Consequently, point 0 on the transverse axis represents the midpoint between G1 and G2. Utilizing our QPS scheme, we continuously and simultaneously measure the distances to both ground stations, resulting in traces for two scenarios. We then compare these measured positioning traces with ideal positioning traces, as illustrated in Figure 7. If the plane flies away along with the perpendicular bisector of G1 and G2, we can obtain its trace using our QPS scheme (measured positioning trace) and compare with the ideal positioning trace, as depicted in Figure 7a. Similarly, if the plane flies away from G1 along a circle centered on G2, we can also easily obtain its trace using our QPS scheme (measured positioning trace) and compare it with the ideal positioning trace, as shown in Figure 7b. Figure 7a,b shows clearly that traces measured by our QPS scheme match the ideal positioning trace well, which means our scheme is precise and effective.

**Figure 8:**
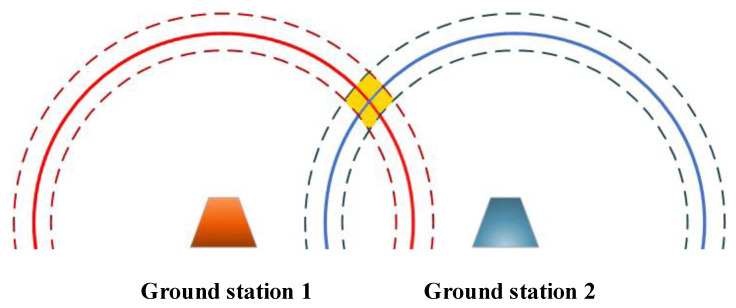
Positioning error zone.

We employ the positioning error as a metric to evaluate the precision of our QPS scheme. The positioning error is illustrated in Figure 8.

If the distances between the plane and G1/G2 are accurately measured, we can determine circles with specific radii and their intersections, enabling us to identify the exact position of the plane. This is well-illustrated by the solid circles in Figure 8. Conversely, if the measurements are not precise, we obtain circles with fluctuating radii and overlapping areas. This overlapping area represents the positioning error of our QPS scheme. In reality, the positioning error stems from distance ranging errors, which result from time interval measurement errors. These time interval measurement errors are influenced by the second-order coherence function, which reveals the presence of quantum entanglement.

Under ideal conditions, disregarding engineering and technical implementation, in a specific scenario of $\eta_{1}=\eta_{2}=0.5$, we can straightforwardly compute the propagation time intervals as $\tau_{1}=\tau_{2}=5.3451\;\mu s$ using Equations (23)–(25). Furthermore, we can approximate the distances as $d_{1}=d_{2}=801.7650$ m by utilizing Equations (26) and (27). To quantify the time interval measurement errors, we utilize the full width at half maximum (FWHM) of the second-order coherence function, denoted as $\Delta \tau_{1}$ and $\Delta \tau_{2}$ for G1 and G2, respectively.

(30) $$\Delta \tau_{1}=\Delta \tau_{2}=\pm \left|\tau_{0.5g_{\max}^{\left(2\right)}(\tau )}-{\tau_{0.5g_{\max}^{\left(2\right)}(\tau )}}^{\prime }\right|=\pm 2.704\mathrm{ps}$$

Then, we can obtain distance measurement errors by

(31) $$\Delta d_{1}=\Delta d_{2}=c\Delta \tau_{1}=\pm 8\times 10^{-4}m$$

In Figure 8, the bright-yellow region represents the positioning error zone associated with our QPS scheme. We can regard it as an approximation rectangle, and we utilize its area to quantify positioning error as

(32) $$E=\left|\Delta d_{1}\right|\times \left|\Delta d_{2}\right|=6.4\times 10^{-7}m^{2}$$

Given the operational distances involved, the positioning error is negligible. In other words, our QPS scheme demonstrates a high degree of precision.

#### 4.3.2. Impact of Key Parameter

(1) Impact of the transmissivity η

**Figure 9:**
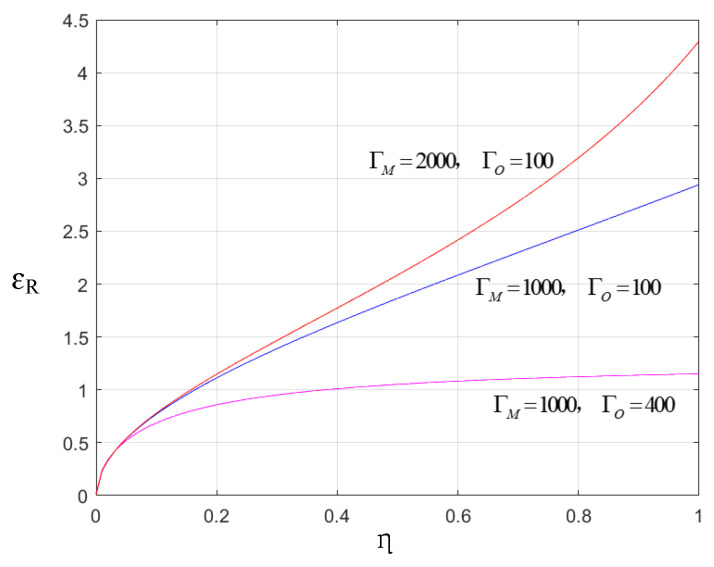
$\varepsilon_{R}$ versus η under different $\Gamma_{M}$ and $\Gamma_{O}$.

The microwave photons emitted from the transmitter undergo reflection by the aircraft, with their number being influenced by the transmissivity η. These photons then arrive at the converter at the receiver, where they undergo microwave-to-optical conversion and are transformed into optical frequency photons. The number of these converted photons is influenced by the conversion efficiency. Consequently, the entanglement in the receiver may be impacted by both the transmissivity η and conversion efficiency. Based on Equations (19)–(22), we have plotted the variation trends of $\varepsilon_{R}$ versus η under three different parameters of $\Gamma_{M}$ and $\Gamma_{O}$, as shown in Figure 9.

**Figure 10:**
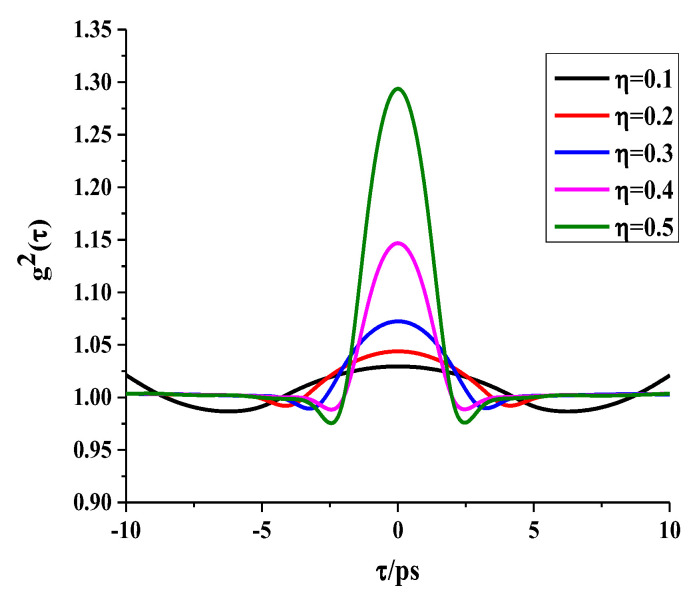
$g^{\left(2\right)}(\tau )$ under different η.

These photons undergo Hanbury Brown–Twiss (HBT) pattern coincidence measurement with local idle photons. Therefore, the number of optical frequency photons at the receiving end is affected by both the transmissivity η and photon conversion efficiency, which, in turn, affects the second-order coherence function $g^{\left(2\right)}(\tau )$. According to Equations (19)–(23), we plot the variances of $g^{\left(2\right)}(\tau )$ under different transmissivity η levels, as seen in Figure 10.

In Figure 10, it is evident that a smaller value of η corresponds to a smaller value of $g_{\max}^{\left(2\right)}(\tau )$. Additionally, it is clear that the full width at half maximum (FWHM) of $g^{\left(2\right)}(\tau )$ broadens as η increases, indicating a larger time interval measurement error Δτ. If distance ranging errors of both ground stations increase, this would result in a larger positioning error for our QPS scheme. However, fortunately, the degradation of the scheme’s performance is minimal, as demonstrated in Figure 10. This reveals that our QPS scheme is robust and stable.

(2) Impact of the photon conversion efficiency

For the purpose of analyzing the impact of photon conversion efficiency, the transfer function that describes the conversion from input microwave photons to output optical photons for cavity electro-opto-mechanic converters is given as follows [38,42,43]:

(33) $$A=-2\sqrt{\frac{\kappa_{m}^{\mathrm{ext}}}{\kappa_{m}}\frac{\kappa_{o}^{\mathrm{ext}}}{\kappa_{o}}}\frac{\sqrt{\Gamma_{m}\Gamma_{o}}}{\gamma_{m}+\Gamma_{o}+\Gamma_{m}}$$

The photon conversion efficiency is defined as the ratio of the number of coherently converted output photons to the number of input photons, and it can be mathematically expressed as follows [38,42,43]:

(34) $$\beta ={\left|A\right|}^{2}=4\eta_{m}\eta_{o}\frac{\Gamma_{m}\Gamma_{o}}{{\left(\gamma_{m}+\Gamma_{o}+\Gamma_{m}\right)}^{2}}$$

where $\eta_{m}=\kappa_{m}^{\mathrm{ext}}/\kappa_{m}$ and $\eta_{o}=\kappa_{o}^{\mathrm{ext}}/\kappa_{o}$ denote the conversion efficiency between the input/output and the stable cavity field.

**Figure 11:**
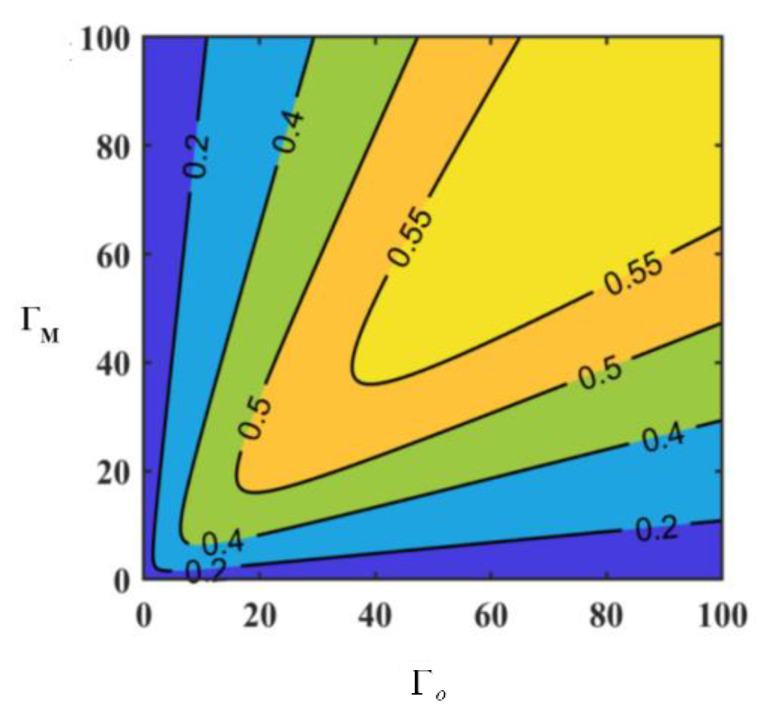
The relationship between microwave–optic conversion efficiency and cooperativity parameters.

We simulated the relationship between photon conversion efficiency and cooperativity parameters, and the results are depicted in Figure 11. The photon conversion efficiency curves exhibit symmetry with respect to the line of $\Gamma_{m}=\Gamma_{o}$, and the photon conversion efficiency reaches its maximum value only when the coupling matching condition $\Gamma_{m}=\Gamma_{o}$ is satisfied.

A reduction in photon conversion efficiency can lead to a decrease in the number of photons reaching the receiving end, producing an effect similar to that of decreased transmissivity. In specific applications of quantum positioning, a higher photon conversion efficiency indicates higher detection sensitivity. When combined with efficient microwave single-photon detectors, receivers based on cavity electro-opto-mechanic converters are anticipated to achieve ultra-high sensitivity, capable of detecting single microwave photons.

## 5. Conclusions

In this paper, we propose a QPS scheme based on M–O entanglement prepared by an EOM converter. With precise distances measurement by ground stations G1 and G2, we can identify the position of the plane by checking intersections of circles. Under ideal conditions, disregarding engineering and technical implementation difficulties, this scheme can achieve extremely high ranging and positioning accuracy, along with excellent stability and robustness. Based on the analysis of key parameters, reductions in transmissivity and photon conversion efficiency will lead to varying degrees of decline in entanglement, ranging and positioning accuracy. Enhancing microwave single-photon detection capability and improving the photon conversion efficiency of EOM converters are crucial goals that urgently need to be addressed for this scheme.

## Figures and Tables

**Table 1:** Denotations of main parameters in cavity.

	Microwave Cavity	Mechanical Oscillator	Optical Cavity
Cavity/vibration mode	${\hat{C}}_{M}$	$\hat{m}$	${\hat{C}}_{O}$
Cavity/vibration mode frequency	$\omega_{M}$	$\omega_{m}$	$\omega_{O}$
Damping rate	$\kappa_{M}$	γ	$\kappa_{O}$
Driving mode frequency	$\omega_{d,M}$	—	$\omega_{d,O}$
Detuning of frequency	$\Delta_{M}$	—	$\Delta_{O}$

**Table 2:** Parameter settings of EOM converter.

	Microwave Cavity	Mechanical Oscillator	Optical Cavity
Frequency	$\frac{\omega_{M}}{2\pi }=$ 6.982 GHz	$\frac{\omega_{m}}{2\pi }=$ 32.5 MHz	$\frac{\omega_{O}}{2\pi }=$ 282 THz
Driving power	$P_{M}=35$ mW	—	$P_{O}=5$ mW
Coupling rate	$\frac{g_{M}}{2\pi }=10.14$ Hz	—	$\frac{g_{O}}{2\pi }=289.75$ Hz
Damping rate	$\kappa_{M}=0.2\omega_{m}$	$\gamma =\omega_{m}/Q$	$\kappa_{O}=0.1\omega_{m}$
Detuning of frequency	$\Delta_{M}=\omega_{m}$	—	$\Delta_{O}=-\omega_{m}$

## Data Availability

The raw data supporting the conclusions of this article will be made available by the authors on requests.

## Appendix A

The expressions of $A_{j}$, B, $C_{j}$ (j=M,O)

By applying quantum Langevin equations to involved cavity modes and substituting several variables as Ref. [38] did, parameters $A_{j}$, B, $C_{j}$ (j=M,O) can be expressed in terms of cooperativity parameter $\Gamma_{j}$ (j=M,O) as

(A1) $$A_{M}=\frac{1-(\Gamma_{M}+\Gamma_{O})}{1+(\Gamma_{M}-\Gamma_{O})}$$

(A2) $$A_{O}=\frac{1+(\Gamma_{M}+\Gamma_{O})}{1+(\Gamma_{M}-\Gamma_{O})}$$

(A3) $$B=\frac{2\sqrt{\Gamma_{M}\Gamma_{O}}}{1+(\Gamma_{M}-\Gamma_{O})}$$

(A4) $$C_{M}=\frac{2i\sqrt{\Gamma_{M}}}{1+(\Gamma_{M}-\Gamma_{O})}$$

(A5) $$C_{O}=\frac{2i\sqrt{\Gamma_{O}}}{1+(\Gamma_{M}-\Gamma_{O})}$$

## Appendix B

Deductions of Equations (5)–(7)

Given the correlations between fluctuation operators as

(A6) $$\langle \delta {\hat{C}}_{i}^{†}\delta {\hat{C}}_{j}\rangle ={\bar{n}}_{i}\delta_{ij}$$

(A7) $$\langle \delta {\hat{C}}_{i}\delta {\hat{C}}_{j}^{†}\rangle =\left({\bar{n}}_{i}+1\right)\delta_{ij}$$

(A8) $$\langle \delta {\hat{C}}_{i}\delta {\hat{C}}_{j}\rangle =\langle \delta {\hat{C}}_{i}^{†}\delta {\hat{C}}_{j}^{†}\rangle =0$$

where $\delta {\hat{C}}_{i},\delta {\hat{C}}_{j}=\delta {\hat{C}}_{M,intra},\delta {\hat{C}}_{O,intra},\delta {\hat{C}}_{m}$, ${\bar{n}}_{i}={\bar{n}}_{M,T_{E}},{\bar{n}}_{O,T_{E}},{\bar{n}}_{m,T_{E}}$, and $\delta_{ij}=\left\{\begin{array}{l} 1,i=j\\ 0,i\neq j \end{array}\right.$, we have

(A9) $$\begin{array}{cc} {\bar{N}}_{M} & =\langle {\hat{M}}^{†}\hat{M}\rangle \\ & =\langle \left(A_{M}^{*}\delta {\hat{C}}_{M,\mathrm{intra}}^{†}-B^{*}\delta {\hat{C}}_{O,\mathrm{intra}}-C_{M}^{*}\delta {\hat{C}}_{m}^{†}\right)\left(A_{M}\delta {\hat{C}}_{M,\mathrm{intra}}-B\delta {\hat{C}}_{O,\mathrm{intra}}^{†}-C_{M}\delta {\hat{C}}_{m}\right)\rangle \\ & ={\left|A_{M}\right|}^{2}\langle \delta {\hat{C}}_{M,\mathrm{intra}}^{†}\delta {\hat{C}}_{M,\mathrm{intra}}\rangle -A_{M}^{*}B\langle \delta {\hat{C}}_{M,\mathrm{intra}}^{†}{\hat{C}}_{O,\mathrm{intra}}^{†}\rangle -A_{M}^{*}C_{M}\langle \delta {\hat{C}}_{M,\mathrm{intra}}^{†}\delta {\hat{C}}_{m}\rangle \\ & -B^{*}A_{M}\langle \delta {\hat{C}}_{O,\mathrm{intra}}\delta {\hat{C}}_{M,\mathrm{intra}}\rangle +{\left|B\right|}^{2}\langle \delta {\hat{C}}_{O,\mathrm{intra}}\delta {\hat{C}}_{O,\mathrm{intra}}^{†}\rangle +B^{*}C_{M}\langle \delta {\hat{C}}_{O,\mathrm{intra}}\delta {\hat{C}}_{m}\rangle \\ & -C_{M}^{*}A_{M}\langle \delta {\hat{C}}_{m}^{†}\delta {\hat{C}}_{M,\mathrm{intra}}\rangle +C_{M}^{*}B\langle \delta {\hat{C}}_{m}^{†}\delta {\hat{C}}_{O,\mathrm{intra}}^{†}\rangle +{\left|C_{M}\right|}^{2}\langle \delta {\hat{C}}_{m}^{†}\delta {\hat{C}}_{m}\rangle \\ & ={\left|A_{M}\right|}^{2}\langle \delta {\hat{C}}_{M,\mathrm{intra}}^{†}\delta {\hat{C}}_{M,\mathrm{intra}}\rangle +{\left|B\right|}^{2}\langle \delta {\hat{C}}_{O,\mathrm{intra}}\delta {\hat{C}}_{O,\mathrm{intra}}^{†}\rangle +{\left|C_{M}\right|}^{2}\langle \delta {\hat{C}}_{m}^{†}\delta {\hat{C}}_{m}\rangle \\ & ={\left|A_{M}\right|}^{2}{\bar{n}}_{M,T_{E}}+{\left|B\right|}^{2}\left({\bar{n}}_{O,T_{E}}+1\right)+{\left|C_{M}\right|}^{2}{\bar{n}}_{m,T_{E}} \end{array}$$

Equation (A9) yields Equation (5), and Equations (6) and (7) can be yielded by similar operations.

$\varepsilon_{11}$ and $\varepsilon_{21}$ can be easily obtained. As for $\varepsilon_{12}$ and $\varepsilon_{22}$, they can be also yielded by similar operations below.

(A10) $$\begin{array}{cc} {\bar{N}}_{MR} & =\langle {\hat{M}}_{R}^{†}{\hat{M}}_{R}\rangle \\ & =\langle \left(\sqrt{\eta }{\hat{M}}^{†}+\sqrt{1-\eta }\delta {\hat{C}}_{B,T_{B}}^{†}\right)\left(\sqrt{\eta }\hat{M}+\sqrt{1-\eta }\delta {\hat{C}}_{B,T_{B}}\right)\rangle \\ & =\eta \langle {\hat{M}}^{†}\hat{M}\rangle +\left(1-\eta \right)\langle \delta {\hat{C}}_{B,T_{B}}^{†}\delta {\hat{C}}_{B,T_{B}}\rangle \\ & =\eta {\bar{N}}_{M}+(1-\eta ){\bar{n}}_{B,T_{B}} \end{array}$$

(A11) $$\begin{array}{cc} {\bar{N}}_{OR} & =\langle {\hat{O}}_{R}^{†}{\hat{O}}_{R}\rangle \\ & =\langle \left(B^{*}{\hat{M}}_{R}+A_{O}^{*}\delta {\hat{C}}_{OR,intra}^{†}-C_{O}^{*}\delta {\hat{C}}_{mR}\right)\left(B{\hat{M}}_{R}^{†}+A_{O}\delta {\hat{C}}_{OR,intra}-C_{O}\delta {\hat{C}}_{mR}^{†}\right)\rangle \\ & ={\left|B\right|}^{2}\langle {\hat{M}}_{R}{\hat{M}}_{R}^{†}\rangle +{\left|A_{O}\right|}^{2}\langle \delta {\hat{C}}_{OR,intra}^{†}\delta {\hat{C}}_{OR,intra}\rangle +{\left|C_{O}\right|}^{2}\langle \delta {\hat{C}}_{mR}\delta {\hat{C}}_{mR}^{†}\rangle \\ & ={\left|B\right|}^{2}({\bar{N}}_{MR}+1)+{\left|A_{O}\right|}^{2}{\bar{n}}_{OR,T_{E}}+{\left|C_{O}\right|}^{2}({\bar{n}}_{mR,T_{E}}+1) \end{array}$$

What should be noticed is that $\delta {\hat{C}}_{OR,intra}$ and $\delta {\hat{C}}_{mR}$ in receiver are in the same states as their counterparts in transmitter, and we can obtain

(A12)
$$\begin{array}{cc} \langle {\hat{O}}_{R}^{†}\hat{O}\rangle & =\langle \left(B^{*}{\hat{M}}_{R}+A_{O}^{*}\delta {\hat{C}}_{OR,\mathrm{intra}}^{†}-C_{O}^{*}\delta {\hat{C}}_{mR}\right)\left(B\delta {\hat{C}}_{M,\mathrm{inta}}^{†}+A_{O}\delta {\hat{C}}_{O,\mathrm{intra}}-C_{O}\delta {\hat{C}}_{m}^{†}\right)\rangle \\ & =\langle \left(B^{*}\left(\sqrt{\eta }\hat{M}+\sqrt{1-\eta }\delta {\hat{C}}_{B,T_{B}}\right)+A_{O}^{*}\delta {\hat{C}}_{OR,\mathrm{intra}}^{†}-C_{O}^{*}\delta {\hat{C}}_{mR}\right)\left(B\delta {\hat{C}}_{M,\mathrm{intra}}^{†}+A_{O}\delta {\hat{C}}_{o,\mathrm{intra}}-C_{o}\delta {\hat{C}}_{m}^{†}\right)\rangle \\ & =\langle \left(\sqrt{\eta }B^{*}\hat{M}+A_{O}^{*}\delta {\hat{C}}_{OR,\mathrm{intra}}^{†}-C_{O}^{*}\delta {\hat{C}}_{mR}\right)\left(B\delta {\hat{C}}_{M,\mathrm{intra}}^{†}+A_{O}\delta {\hat{C}}_{O,\mathrm{intra}}-C_{o}\delta {\hat{C}}_{m}^{†}\right)\rangle \\ & =\sqrt{\eta }B\langle \hat{M}\hat{O}\rangle +{\left|A_{o}\right|}^{2}{\bar{n}}_{O,T_{E}}+{\left|C_{o}\right|}^{2}\left({\bar{n}}_{m,T_{E}}+1\right) \end{array}$$

Hence, $\varepsilon_{12}$ and $\varepsilon_{22}$ can be also yielded easily.
